# Evaluation of Brazilian web site information on allergic rhinitis

**DOI:** 10.1016/S1808-8694(15)31262-3

**Published:** 2015-10-20

**Authors:** Leonardo Victor España Rueda Silva, João Ferreira de Mello, Olavo Mion

**Affiliations:** 1 Medical School, Studies under course, University of Sao Paulo.; 2 Collaborating Professor, Discipline of Otorhinolaryngology, Medical School, University of Sao Paulo.; 3 Collaborating Professor, Discipline of Otorhinolaryngology, Medical School, University of Sao Paulo. Division of Clinical Otorhinolaryngology, Hospital das Clínicas, Medical School, University of Sao Paulo.

**Keywords:** allergic rhinitis, medical ethics, internet

## Abstract

Nowadays, the World Wide Web (Internet) is an information source for non-experts and physicians. **Aim**: To evaluate, based on ethical principles, Brazilian web sites information about “allergic rhinitis”. Allergic rhinitis is a very common disease, effecting more than 10% of the general population, leading to decrease in quality of life. **Study design:** review. **Material and Method**: We performed the evaluation of 173 Brazilian web sites, which were obtained from four search engines (Google, Yahoo, AltaVista and Radar Uol). The web sites were evaluated according to the Manual of Ethical Principles, Regional Council of Medicine of the state of Sao Paulo (CREMESP), regarding transparency, honesty, quality, privacy, medical ethics, informed consent, responsibility and origin. **Results**: Among the analyzed web sites, 149 (86.1%) were not in accordance with the Manual of Ethical Principles of Regional Council of Medicine of the state of Sao Paulo (CREMESP). According to the analyzed items, the irregularities that were found were quality (84.4%), privacy (46.2%), honesty (18.5%), informed consent (15.6%), responsibility and origin (13.9%), transparency (12.1%), medical ethics (2.3%). There was inaccurate information in 24.3% of the analyzed sites. **Conclusions**: The majority of the websites regarding allergic rhinitis are not in accordance with the ethical principles of CREMESP. In general, the quality of a great part of the Brazilian web sites that address “allergic rhinitis”, and the quality of the information disseminated by them, are insufficient to satisfy doctors and patients.

## INTRODUCTION

The Internet started in 1969 in the United States to interconnect research labs and it has constantly progressed since its creation to our current time. Among the factors that contributed to this progression, we can include the creation of the World Wide Web in 1992, which facilitated the access to information by users; the creation of electronic mail that enabled messages to be sent to any user whose address is known, regardless of the distance or the location, in addition to the creation of search web sites and browser programs. Owing to easy access to internet, the number of users has grown constantly all over the world. ^1^

In Brazil, the internet has had a significant growth. In January 2003, Brazil had 7.3 million active home users; in October 2004, it is estimated that there were 18.6 million home users with access to the internet, and there are 11.6 million active users. ^2^

Internet has facilitated the dissemination of scientific information among healthcare professionals in different ways, providing free bibliographical reviews, communication of the contents of journals of different specialties, and facilitating the acquisition of medical equipment and didactic material, in addition to promoting the exchange of information through discussion groups, videoconferences and other valuable contributions to the medical area.

Moreover, owing to the facility to get information transmitted through the web through free access providers and owing to the existence of free internet access points, the lay people can easily learn about prevention, diagnosis and treatment of diseases, which help them complement the information given by the physician.

The dissemination of information, provision of services and sales of medical products through the internet has potential to promote healthcare, but it can also cause damage to internet users and consumers by means of communication of imprecise information, inappropriate use of users’ personal data, dishonest advertising of products, etc.

In Brazil, there is no specific legislation to regulate the use of Internet or electronic mail. In view of that, the Regional Council of the State of Sao Paulo (CREMESP), aware of the need to self-regulate the medical sector to define minimal standards of quality, safety and reliability of medical and health-related websites, has enacted on March 9, 2001 a resolution with four articles for the use of internet by medicine and health-related web sites. As an annex, they published the Manual of Ethical Principles for Medicine and Healthcare Web Sites. ^3^

In this manual, the quality of the website is assessed according to the following parameters: transparency, honesty, quality of information, free and informed consent, privacy, medical ethics, responsibility and origin.

Allergic rhinitis affects nearly 10 to 20% of the world population and over 15% to 25% of children and adolescents. Moreover, allergic rhinitis has a major social-economic impact, estimating that about 60% absences to work are directly related with upper respiratory allergy, and a significant part of them are caused by allergic rhinitis. ^4^

Owing to the high prevalence and the major social-economic impact of allergic rhinitis, in addition to the abundance of information about the disease in Brazilian sites in the internet, the present study intended to assess the ethical and quality parameters of the Brazilian web sites that communicated information about allergic rhinitis, following the principles of assessment advocated by the Manual of Ethical Principles for Medicine and Healthcare Web Sites

## MATERIAL AND METHOD

To choose the web sites that were assessed in the present study, we used the four largest search engines in the Brazilian internet: Google, AltaVista, Yahoo and Radar Uol. Between March 17 to 20, 2004 we searched these web sites looking for pages in Brazil that had the key words “allergic rhinitis” and we obtained: 5,220 pages at Google (www.google.com.br), 878 pages at Yahoo do Brasil (www.yahoo.com.br), 1,427 pages at AltaVista do Brasil (br.altavista.com) and 1,453 pages at Radar Uol (radaruol.uol.com.br).

It was pre-determined that we would only assess the first 100 results obtained from each site given that the first results obtained from search sites are the most accessed by users. ^5^ This was the case in this large number of web sites about allergic rhinitis that we found.

We excluded results that were retrieved from more than one search page and the sites that did not specifically address the topic allergic rhinitis, such as specialization tests in Otorhinolaryngology, sites about topical dermatitis and asthma, printing advertisements, courses, etc. Moreover, we excluded the web sites that presented technical problems upon access between March 17 and 19, 2004 and after one more attempt on April 20, 2004. To assess the information on allergic rhinitis in Brazilian sites we selected the first 100 results obtained from each of the search engines: Google, Yahoo, Altavista and Radar Uol, totaling 400 results. We excluded 20 web sites that had technical problems on the 2 days we tried to access them (5% of the total), 184 web sites that had already been found by other search mechanism (46% of the total) and 23 sites that did not specifically address allergic rhinitis (5.75% of the total results). Thus, after the selection, we identified 173 sites (43.25% of the initial sample) to be completely investigated and analyzed.

Each site was assessed according to the first article of the Manual of Ethical Principles for Medicine and Healthcare concerning the items and sub items: transparency (assessed related to its own contents and identification of the person responsible for the site), honesty concerning the site objectives, quality of information (assessed concerning precision, updating and appropriateness of language and scientific grounding, with authors and bibliographical references), free informed consent, privacy of information provided by the web site, medical ethics (in which we tried to find situations that disrespected the medical ethics), responsibility and origin (assessing what was explicit by the responsible person about the site, whether there was a way to contact him/her and if the web site had tools for the users to express their opinions).

The precision of information on allergic rhinitis was assessed according to the Treaty of Otorhinolaryngology, Brazilian Society of Otorhinolaryngology. ^6^

Each item was only considered appropriate if all sub items were classified as such, and the site was considered appropriate only if all items were classified as such.

## RESULTS

Allergic rhinitis is one of the healthcare topics that has more information about it on the internet. For example, in a study performed between March 17 and 20, 2004 in Brazilian web sites using the words allergic rhinitis, we found 5,220 pages at Google do Brasil, 878 pages at Yahoo do Brasil, 1,427 pages at AltaVista do Brasil, and 1,453 pages at Radar Uol.

To assess the information about allergic rhinitis in Brazilian sites we selected the first 100 sites given by the search mechanisms that were studied: Google, Yahoo, Altavista and Radar Uol, totaling 400 results. Out of this result, 20 sites had technical problems on the two days we tried to access them (5% of the total results), 184 sites had already been found in other search mechanism (46% of the total) and 23 sites did not specifically address the topic of allergic rhinitis (5.75% of the total). Thus, after such selection, we chose 173 sites (43.25% from the initial sample) to be completely analyzed and assessed.

Among the 173 selected sites, 11 (6.36%) were pages of societies recognized by the Brazilian Medical Association (AMB), 21 (12.14%) were clinical medical pages, 7 (4.05%) were hospital pages, 22 (12.72%) were pharmaceutical company pages, 68 (39.31%) were pages from other private companies, 18 (10.40%) were private physician and other healthcare professional pages, 4 (2.31%) were private pages of people that were not with the healthcare area, 12 (6.94%) were pages of non-profitable societies, and in 10 (5.78%) pages it was not possible to identify the person responsible for the presented information.

Among the 173 analyzed sites, 146 (84.40%) contained information to the lay people, whereas 27 sites (15.60%) had as main target audience physicians and other healthcare professionals.

Out of the selected web sites, 91 (52.60%) had product advertisements (dehumidifiers, air purifiers, mite solutions, etc.), drugs (allopathic, homeopathic, herbal, orthomolecular complexes, floral drugs, etc.) or services (home vaccination, acupuncture, visits with immunologists and Otorhino- laryngologists, etc.) to treat allergic rhinitis. However, 82 (47.40%) of the web sites did not have this type of ad.

Among the assessed sites, we classified 152 (87.9%) sites as appropriate concerning the item transparency, whereas 21 (12.1%) were classified as inappropriate. The classification was based on the fact that 154 (89%) of the web sites and 19 (11%) had or had not, respectively, clearly stated their purposes - educational or commercial - oriented to sell advertisement space, products, services, personalized medical care, assistant or counseling. Moreover, 157 (90.8%) correctly presented the name of the responsible people, sponsors or organizers of the site, which was not seen in 16 (9.2%) sites (Table 1).

**Table 1:** Classification of analyzed sites according to the Manual of Ethical Principles for Medical and Healthcare Sites.

CATEGORY	Compliant		Non-compliant		Not applicable	
	N°	%	N°	%	N°	%
Transparency	152	87,9	21	12,1	0	0,0
Purpose	154	89,0	19	11,0	0	0,0
Responsibility	157	90,8	16	9,2	0	0,0
Honesty	141	81,5	32	18,5	0	0,0
Quality	27	15,6	146	84,4	0	0,0
Precision	131	75,7	42	24,3	0	0,0
Update	71	41,0	102	59,0	0	0,0
Language	132	76,3	41	23,7	0	0,0
Scientific	87	50,3	86	49,7	O	0,0
grounding - author Scientific	27	15,6	128	74,0	18	10,4
grounding - bibliography Consent	83	48,0	27	15,6	63	36,4
Privacy	30	17,3	80	46,2	63	36,4
Medical Ethics	169	97,7	4	2,3	0	0,0
Responsibility	149	86,1	24	13,9	0	0,0
Identification	158	91,3	15	8,7	0	0,0
Contact	170	98,2	3	1,7	0	0,0
Interaction with user	163	94,2	10	5,8	0	0,0
All analyzed items	24	13,9	149	86,1	0	0,0

We observed that 141 (81.5%) were compliant and 32 (18.5%) were not compliant concerning honesty of information, that is, clear objective of the educational or scientific communicated content (Table 1).

Upon analyzing the items responsible for the quality of the information (precision, updating, language, authorship and scientific grounding), we classified 27 sites (15.6%) as compliant and 146 (84.4%) as non-compliant (Tables 1 and 2).

**Table 2:** Quality of web sites and quality of information concerning type of responsible person.

Type of responsible people	Compliant sites *		Appropriate quality+		Total §
	Nº	%	Nº	%	Nº
Societies	2	18,18	5	45,45	11
recognized by AMB					
Medical clinics	4	19,05	4	19,05	21
Hospitals	1	14,29	1	14,29	7
Pharmaceutical companies	0	0,00	0	0,00	22
Other companies	6	8,82	12	17,65	68
Private physicians	3	16,67	3	16.67	18
Private non-medical professionals	0	0,00	0	0,00	4
Non-profitable entities	0	0,00	0	0.00	12
Not identified	0	0,00	2	20,00	10

Total	16	9,25	27	15,61	173

* Sites compliant with the items of Manual of Ethical Principles for Medical and Healthcare Sites

+ Sites compliant with all items concerning quality of information (precision, updating, language, scientific foundation - author and bibliography)

§ Total of sites with the same type of responsible person

We observed that 131 (75.7%) of the web sites presented precise information about allergic rhinitis, whereas 42 (24.3%) had imprecise information about allergic rhinitis (Table 1, 2 and 3).

**Table 3:** Imprecision of information found in Brazilian sites that communicate information about allergic rhinitis and their respective corrections.

IMPRECISION	CORRECTION
- "Exercise, emotional factors, strong smells, drugs, ozone, cigarettes, chlorine are triggers of allergic rhinitis (allergens)"	- Some of these factors may aggravate allergic rhinitis by irritating the nasal mucosa, but none of them are triggering effects of allergic rhinitis
- "Allergic rhinitis or hay fever"	- Only seasonal or intermittent allergic rhinitis may be called hay fever
- "Ipatropium Bromide is a drug used to treat allergic rhinitis"	- Ipatropium bromide for nasal use has been withdrawn from the Brazilian market (it is no longer used for allergic rhinitis)
- "Antibiotics and purified allergic extracts, homeopathy, acupuncture, herbs and mushrooms, orthomolecular kits are effective in the treatment of allergic rhinitis"	- There is no scientific confirmation of the efficacy of these items in the treatment of allergic rhinitis
- "IgE produces chemical substances that lead to local inflammation that causes the symptoms of allergic rhinitis"	- IgE binds to receptors of mast cells that release chemical substances that are stored, such as histamine.

As to updating, 102 (59%) of the sites presented incorrect information, such as absence of date of the last update or they contained dated information about allergic rhinitis. At the same time, 71 (41.0%) sites were appropriate concerning updating (Table 1 and 2).

As to the language used by the sites, 41 (23.7%) of the analyzed sites presented inappropriate elements such as conceptual mistakes about rhinitis or they presented grammar mistakes or language not directed to the target audience. However, 132 (76.3%) were appropriate concerning language parameters (Table 1 and 2).

Among the analyzed sites, 86 (49.7%) had no qualified professional appointed as the responsible person (author or collaborator), but 87 (50.3%) sites were within the advocated norms (Table 1 and 2).

We observed that in 128 (74%) of the sites there was no scientific grounding for the communicated information, giving no clarification about the origin of information, such as trials. research studies, protocols, consensus or clinical practice. In 18 (10.4%), it was not possible to assess the element scientific grounding because they were interviews with healthcare professionals. However, 27 (15.6%) sites were in compliance with the elements of scientific grounding (Table 1 and 2).

Among the sites that had quality of information, there were 11 sites of societies recognized by AMB (45.5% of the sites of the category), 4 medical clinics (19.05%), 1 hospital site (14.29% of the category), 12 of other companies (17.65% of the category), 3 private doctor sites (16.67% of the category), 2 sites with non-identified responsible people (20.0% of the category) (Table 2).

We also observed that 83 (48%) of the sites were compliant with the principle of free and informed consent (clearly explaining who collected the data, the reasons for it, and how data were used and shared). However, there were 27 (15.6%) web sites non-compliant with this aspect. Moreover, 63 (36.4%) did not require any information from the users, meaning that the compliance with free and informed consent was not applicable (Table 1).

We classified 30 sites (17.3%) as appropriate concerning applied privacy. However, 80 sites (46.2%) were non compliant with this item. Moreover, 63 (36.4%) sites did not require personal information about the users, which did not allow the assessment of site privacy policies (Table 1).

To analyze the compliance with medical ethics of the analyzed site, we searched for situations that did not comply with these principles. Thus, in 4 sites (2.3%), we found medical ethical non-compliances, such as identifiable picture of patients, internet-based visits, and statements informing that the site responsible people would not be held accountable for any damage caused to the user as a result of the communicated information (Table 1).

As to responsibility and origin, 149 sites (86.1%) were classified as appropriate and 24 (13.9%) as inappropriate. Within such item, we assessed whether there had been identification of the responsible people (anyone or any public or private institution that could be legally and ethically responsible for the information, products and medical and health services promoted through the internet), which amounted to 158 sites (91.3%), but there were 15 sites (8.7%) in which it was not found. We also assessed the possibility to find the responsible people, which was observed in 170 (98.3%) sites, but not in 3 (1.7%) sites. We also assessed whether the site had tools that would allow the user to have an opinion, complaint or question. Such tools were present in 163 (94.2%) sites, but absent in other 10 (5.8%) sites (Table 1).

As to all analyzed items (objective, quality of information, free informed consent, privacy of information provided by the site, medical ethics, responsibility and origin), only 16 sites (9.25% of the total analyzed sites) were in accordance with the Manual of Ethical Principles for Medical and Healthcare Sites (Table 1 and 2). Among those sites, there are 2 sites of societies recognized by AMB (18.18% of those belonging to this category), 4 medical clinic sites (19.05% of the category), 1 hospital site (14.29% of the category), 6 sites of non-pharmaceutical private companies (8.82% of the category) and 3 private physician sites (16.67% of the category) (Table 2).

Among the web sites that were in compliance with Manual of Ethical Principles for Medical and Healthcare Sites, 12 were directed to lay people (18.52% of the category) and 5 to physicians and other healthcare professionals (8.22% of the category) (Table 2).

## DISCUSSION

Organizations and subjects, when creating and maintaining medical and healthcare web sites, should provide reliable, correct and high quality content, protecting the privacy of citizens and respecting the rules that regulate the professional ethical medical exercise.

Assessing the information about allergic rhinitis in 173 Brazilian web sites concerning the rules of the Manual of Ethical Principles for Medical and Healthcare Sites, we found many cases of non-compliances in the assessed items.

We noticed that quality of information was the item with the highest number of non-compliances. What has impaired the most the quality of information was lack of reference. The absence of references means that users, either lay people or healthcare professionals, can not check the information communicated by the sites and they do not have the opportunity to further learn about the information communicated by the website. Moreover, the lack of references does not provide scientific grounding to the information, which can be only the result of clinical experience of the people providing the information, without any further confirmation.

The quality of information was very much affected because of update of information. In many web sites, there was no reference to date of creation or text updating, which makes the user unsure about the information provided, making people unsure about their timeliness. Another factor that has hindered information update was presence of dated information, especially the information about topical ipatropium bromide, which is no longer available in Brazil by the pharmaceutical industry for nasal use, even though it may still be prepared. Such incorrect data may lead physicians to making incorrect prescriptions to their patients, which can generate questions and uncertainties (Table 3).

Many sites did not clearly state who the author of the information was. This fact prevents the user from directly contacting the author to clarify doubts, give suggestions or even point out any misinformation found.

In some sites, there was incorrect information published (Table 3).

Some mistakes found are, for example, “The cure of allergy lies in environmental control and anti-allergic vaccines” (allergic rhinitis may only be controlled and not cured); “allergens - substances capable of triggering an allergic reaction - such as strong perfumes, cigarette smoke” (perfumes and cigarette smoke are irritants and not allergens); “ozone is a toxic gas responsible for most of the allergic rhinitis episodes” (ozone is not an allergen triggering allergic rhinitis); or “allergic rhinitis, also known as hay fever” (only seasonal allergic rhinitis is known as hay fever). Such mistakes may lead to questioning the credibility of the information and may make people confused. Moreover, the patient that uses the internet to better learn about the disease may disagree with the physician when is based on imprecise information provided by the web (Table 3).

We could find expressions such as “decongestants, that alleviate nasal congestion. These drugs should be used under medical prescription in people with arterial blood hypertension”. Such piece of information encourages self-medication in people that access these web sites, especially patients with normal blood pressure, in this case (Table 3).

Moreover, the communication of treatments not approved by the Medical Federal Council, such as acupuncture, phytotherapy, orthomolecular kits, make the patients search for treatments that are not necessarily effective and may bring financial loss or health complications (Table 3).

In a research study with 22 Brazilian sites that addressed the topic of allergic rhinitis, Balbani et al. (2000) ^7^ found imprecise information in 13.6% of the sites, a number below that of the present study, in a sampling that was similar but broader. Thus, one can assume that if more sites are assessed, we may find even further imprecise information.

The imprecision of information about health topics in the Internet does not seem to be restricted to the topic of allergic rhinitis and Brazilian sites. In a British survey with 63 sites whose topic was celiac disease, 66% of them were classified as having less than 50% accuracy, especially owing to lack of information, but also imprecise information in 15.9% of the sites ^8^. In an assessment of 38 American sites about the topic “bladder cancer”, we observed inaccuracy within at least 6 factors (such as incidence, staging, recurrence, tumor treatment in initial staging and metastatic cancer) in 32% of the sites. ^9^

In some sites, we found incorrect use of language. Among the most common inappropriate findings we can include mistakes of definition, such as that allergens are confused with irritating factors. Moreover, some sites presented many grammar mistakes, such as misspelled rhinitis, plus grammar mistakes (there was one page with nearly 16 mistakes). Such mistakes show lack of attention when providing information through the internet and may lead to users questioning the cultural level of the person responsible for the information and the site.

Privacy was compliant with the expected rules in only 17.3% of the sites. In all the other sites that required information from users, there was no clarity concerning storage and safety mechanisms to prevent inappropriate use of data and users were not aware whether they had access to the file where their personal data were stored to cancel or modify the records. Thus, the user would be unsure whether to provide data or not, such as address and taxpayers’ number, or they could provide information to be misused by the website. In addition, the user did not know how to cancel or modify the information, which could be used even against their will.

Another item that had many variations among the studied websites was lack of free informed consent. One of the most common findings was registration of users without explaining why. Upon registering a user, the sites required information such as zip code, address and taxpayers’ numbers and did not explain how they would use them, and they could have been used for sending mail without the users’ consent or with other purposes that could harm the user who had provided the data. Another non-compliance was the request of personal information (address, taxpayers’ number, etc) from the users to get answers to questions or suggestions.

Honesty was also non-compliant in some sites. Ads of offices or communication of some types of treatment approaches provided in informative texts to the public may lead users to search for the services, without unbiased information for them to come to their own conclusions. In some sites of the pharmaceutical industry, information is provided only about the active principles manufactured by them (and not about the drug class), such as for example desloratadine (and not anti-histaminic), providing only a partial and incomplete view to users, especially lay people who do not have access to information about other types of treatments that could be used to fight against allergic rhinitis.

In some pages, we found inappropriate information about the person responsible for the site. When accessing any of the 15 sites that do not identify the person responsible for the information, the users may doubt the veracity of the information and if there are questions or suggestion, they will not know to whom send them (some sites offer a contact form, but we do not know who the responsible party is). In some analyzed sites, there were no tools to facilitate the contact with the user, and in some cases it may prevent them from asking questions because the process is very complicated.

We also found compliance concerning transparency of information. These elements include lack of clarity about the purpose of the sites - if they were educational or if they had commercial purposes. Thus, the users cannot know which are the factors that could interfere in the communicated information, to judge the partiality of information conveyed in the web site with commercial purposes directed to sales of products or services. Other items that hindered the transparency of sites was no presentation of the name of the responsible person, direct or indirect sponsor or supporter of the web sites, which has also hindered the assessment of partial information, which could be influenced according to the sponsorship or person responsible for maintaining or supporting the site.

Among the 173 analyzed Brazilian sites that contained information on allergic rhinitis, most of them were not in compliance with the Manual of Ethical Principles for Medical and Healthcare Sites. Among the sites that were not in compliance, most of them are directed to lay people and to physicians and healthcare professionals, such as most of the web sites under the societies recognized by AMB, health clinics, hospitals, pharmaceutical companies, and other private medical and non-medical companies and non-profitable institutions and non-identified domains. It shows that the problems concerning transparency, honesty, quality of information, free informed consent, privacy, medical ethics, responsibility and origin, is not restricted to a specific type of site or a specific type of target audience.

Upon observing such results concerning quality of web sites that provide information about allergic rhinitis, we got concerned about the fact that users may be exposed to incorrect information and take measures based on the information they read, leading to financial losses and health damage. In addition, we may assume that such problems are not restricted to sites that address allergic rhinitis, but rather to other sites that address health-related topics, such as hypertension, diabetes, asthma, etc.

In view of these problems, we should take measures to control them. Some measurements that could be taken would be the formation of medical and specialist groups in the internet to assess web sites that have health content. One possible setback is the large number of web sites available on the Brazilian Internet and the constant flow of information available in the web.

Another measurement that could be applied would be the creation of portals dedicated exclusively to health issues of interest to the lay people, with previously assessed information to be found by search mechanisms. These portals could be organized by the Ministry or Secretary of Health, Medical Council or specialty societies, to ensure quality of web sites and information.

We could create web sites with information not only about the lay people, but also about how health care professionals could have access to reliable information about the studied topic, including bibliographical references. A model of site is rhinitisinfo.com^10^, which has precise information about rhinitis directed to specialists or lay people, including the respective bibliographical references.

## CONCLUSION

According to the Manual of Ethical Principles for Medical and Healthcare Sites, the quality of most of the assessed Brazilian web sites that address the topic allergic rhinitis, comprising the information included in them, is insufficient to meet the needs of physicians and patients.

The main non-compliances were in decreasing order: quality of information, privacy, honesty, free informed consent, responsibility, transparency and compliance with medical ethics.

It is mandatory that we take measurements to improve the situation, which could be made by the Secretary of Health, medical societies and other entities.
